# Overexpression and optimization of glutamate decarboxylase in *Lactobacillus plantarum* Taj-Apis362 for high gamma-aminobutyric acid production

**DOI:** 10.1111/1751-7915.12254

**Published:** 2015-03-10

**Authors:** Naser Tajabadi, Ali Baradaran, Afshin Ebrahimpour, Raha A Rahim, Fatimah A Bakar, Mohd Yazid A Manap, Abdulkarim S Mohammed, Nazamid Saari

**Affiliations:** 1 Department of Food Science, Faculty of Food Science and Technology, University Putra MalaysiaSerdang, Selangor, 43400, Malaysia; 2 Department of Cell and Molecular Biology, Faculty of Biotechnology and Biomolecular Sciences, University Putra MalaysiaSerdang, Selangor, 43400, Malaysia; 3 Department of Honey Bee, Animal Science Research Institute of Iran (ASRI)Karaj, Iran

## Abstract

Gamma-aminobutyric acid (GABA) is an important bioactive compound biosynthesized by microorganisms through decarboxylation of glutamate by glutamate decarboxylase (*GAD*). In this study, a full-length *GAD* gene was obtained by cloning the template deoxyribonucleic acid to pTZ57R/T vector. The open reading frame of the *GAD* gene showed the cloned gene was composed of 1410 nucleotides and encoded a 469 amino acids protein. To improve the GABA-production, the *GAD* gene was cloned into pMG36e-*LbGAD*, and then expressed in *Lactobacillus plantarum* Taj-Apis362 cells. The overexpression was confirmed by SDS-PAGE and *GAD* activity, showing a 53 KDa protein with the enzyme activity increased by sevenfold compared with the original *GAD* activity. The optimal fermentation conditions for GABA production established using response surface methodology were at glutamic acid concentration of 497.973 mM, temperature 36°C, pH 5.31 and time 60 h. Under the conditions, maximum GABA concentration obtained (11.09 mM) was comparable with the predicted value by the model at 11.23 mM. To our knowledge, this is the first report of successful cloning (clone-back) and overexpression of the *LbGAD* gene from *L. plantarum* to *L. plantarum* cells. The recombinant *Lactobacillus* could be used as a starter culture for direct incorporation into a food system during fermentation for production of GABA-rich products.

## Introduction

Gamma-aminobutyric acid (GABA), a non-proteinaceous amino acid, is known to be a major inhibitory neurotransmitter in the mammalian brain tissues (Park and Oh, 2007a,b). The health benefits of GABA have been well documented which include anti-stress effects in humans. Gamma-aminobutyric acid also plays an important role in the reduction of anxiety, improve mood and regulating cardiovascular function (Yokoyama *et al*., 2002; Vaiva *et al*., 2004; Cho *et al*., 2007; Tujioka *et al*., 2007; Ma *et al*., 2013). Interest in the production of GABA-rich products has attracted scientists to investigate the role of glutamate decarboxylase (GAD: EC 4.1.1.15) that is a unique pyridoxal enzyme catalysing α-decarboxylation of L-glutamate to GABA (Li and Cao, 2010). Glutamate decarboxylase is found in different groups of microbes, including lactic acid bacteria (LAB), yeast and fungi. Gamma-aminobutyric acid production by various microorganisms has been actively explored (Kono and Himeno, 2000; Komatsuzaki *et al*., 2005; Park and Oh, 2007a,b; Seo *et al*., 2013).

Among the microbes, LAB are of interest to food manufactures as they are categorized as ‘generally regarded as safe’ (GRAS), and have been used in the production of fermented foods such as cheese, yogurt and beverages. Since the biomass of these harmless and food-grade microbes of LAB is ingested regularly by people as natural food components, they are superior candidates for appraisal as used in bacterial cell factories for GABA production and in the food industry. To date, LABs such as *Lactobacillus brevis* (Kim *et al*., 2007; 2009; Park and Oh, 2007a,b), *Lactobacillus delbrueckii* (Siragusa *et al*., 2007), *Lactobacillus plantarum* (Park and Oh, 2004), *Lactobacillus paracasei* (Siragusa *et al*., 2007) and *Lactococcus lactis* (Siragusa *et al*., 2007) have been identified capable of producing GABA. The corresponding genes encoding *GAD* from *L. plantarum* (Park and Oh, 2004), *Lactobacillus brevis* OPK-3 (Park and Oh, 2007a,b), *Lactobacillus brevis* BH2 (Park and Oh, 2007a,b) and *Lactobacillus brevis* CGMCC 1306 (Fan *et al*., 2012) have been cloned and expressed in *Escherichia coli*. Furthermore, the core fragments of gadB from *L. lactis*, *L. plantarum* C48, *L. paracasei* PF6 and *L. delbrueckii* subsp. *bulgaricus* PR1 have been cloned and sequenced (Siragusa *et al*., 2007). However, *E. coli* cannot be used as a starter culture in the food system since it is not registered as GRAS. On the other hand, the *GAD* gene from *L. brevis* OPK-3 and *L. brevis* Lb85 was successfully expressed in *Bacillus subtilis* and *Corynebacterium glutamicum* respectively (Park and Oh, 2006; Shi *et al*., 2013). However, these bacteria have a limited use in the food fermentation systems; hence, enhancing GABA production by recombinant *GAD* in GABA-producing LAB could help in the development of a starter culture for diverse food applications.

The present study describes the identification of a full-length *GAD* gene from a novel strain of LAB, *L. plantarum* Taj-Apis362 and cloning and overexpression of *L. plantarum* Taj-Apis362-derived *GAD* gene in *L. plantarum* Taj-Apis362 competent cells. The applicability of genetically engineered *L. plantarum* Taj-Apis362 for the enhancement of GABA production is also reported. To our knowledge, this is the first report of cloning (clone-back) and overexpression of the *LbGAD* gene from *L. plantarum* to *L. plantarum* cells. Finally, optimal fermentation conditions for producing maximum GABA were established using response surface methodology (RSM).

## Results and discussion

### Cloning of GAD gene from L. plantarum Taj-Apis362 and sequence analysis

**Fig 1:**
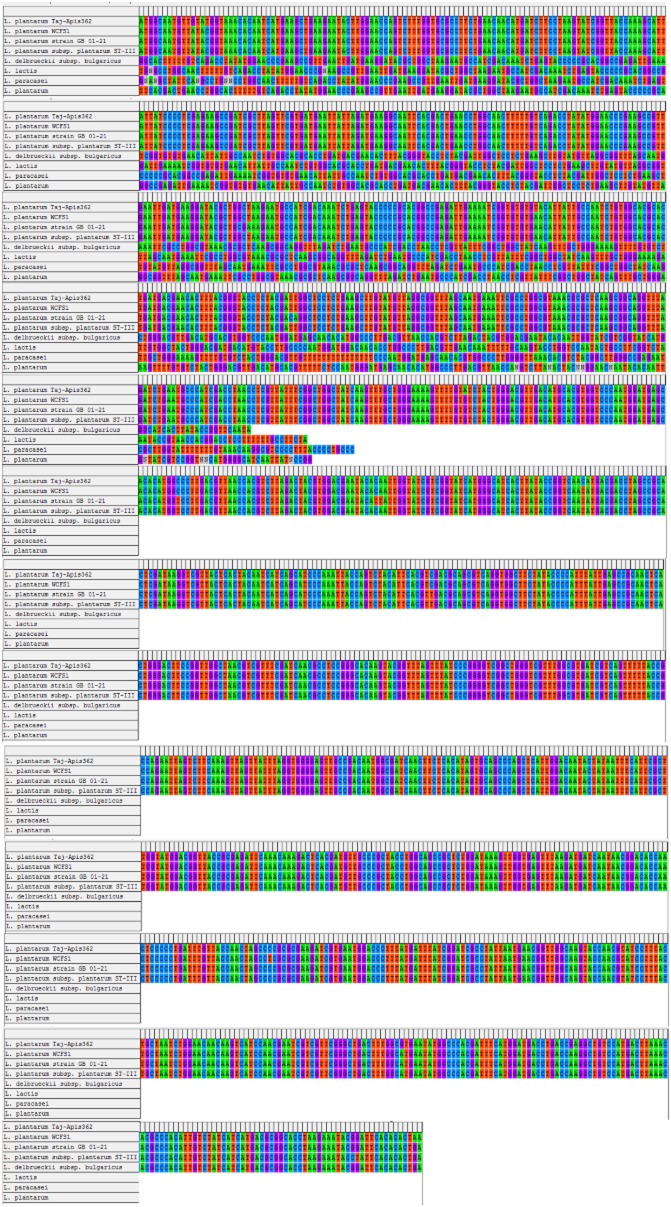
Multiple alignment of *GAD* sequences of *L. plantarum* Taj-Apis362 with *GAD*s from various *Lactobacillus* species. *Lactobacillus plantarum* WCFS1, accession number: AL935263; *L. plantarum* strain GB 01-21 glutamate decarboxylase gene, accession number: JN248358; *L. plantarum* subsp. *plantarum* ST-III, accession number: CP002222; *L. delbrueckii* subsp. *bulgaricus* glutamate decarboxylase gene, accession number: EF174472; *L. lactis* glutamate decarboxylase-like gene, accession number: EF174474; *L. paracasei* glutamate decarboxylase-like gene, accession number: EF174473; *L. plantarum* glutamate decarboxylase-like gene, accession number: EF174475. The asterisks (*) represent identical *GAD* in all sequences in the alignment. The deduced amino acid sequence was analysed using the Clustal W (Tamura *et al*., 2007).

In order to clone the *GAD* gene from *L. plantarum* Taj-Apis362, the polymerase chain reaction (PCR) for the core fragment was performed using primers designed from highly conserved regions of *GAD* as hypothesized by Fan and colleagues (2012). After the first PCR reaction, the PCR product with 1274 bp that matched to the core fragment was cloned into pTZ57R/T vector and sequenced. A complete open reading frame (ORF) encoded a protein (*GAD*) of 469 amino acids with a predicted molecular weight of 53.6 kDa was established. As a comparison, a full-length *GAD* genes were also isolated from *L. brevis* OPK-3 (Park and Oh, 2006), *L. paracasei* (Komatsuzaki *et al*., 2008), *L. lactis* 01-7 (Nomura *et al*., 1999), *L. plantarum* KCTC3015 (Park and Oh, 2004) and *L. brevis* CGMCC 1306 (Fan *et al*., 2012). Komatsuzaki and colleagues (2008) reported that the *GAD* gene consists of 1443 bp encoded a protein of 481 amino acid residues with the predicted molecular mass of 54.3 kDa. In another study, a full-length *GAD* gene was isolated from *L. brevis* BH2, which was composed of 1407 nucleotides and encoded a protein of 468 amino acids with a predicted molecular weight of 53.5 kDa (Kim *et al*., 2007). The deduced amino acid sequence of *GAD* (Fig. 1) from *L. plantarum* Taj-Apis362 was found to have a similarity of 99%, 82%, 67% and 64% with other *GAD* proteins from *L. plantarum* WCFS1, *L. brevis*, *L. lactis* subsp. cremoris MG1363 and *Lactobacillus reuteri* TD1 respectively. Thus, it can be concluded that *GAD* proteins from *L. plantarum* Taj-Apis362 contained highly conserved catalytic domains. The *GAD* gene of *L. plantarum* Taj-Apis362 has been deposited into the GenBank under the accession number of KF770955.

### Overexpression and activity of LbGAD

**Fig 2:**
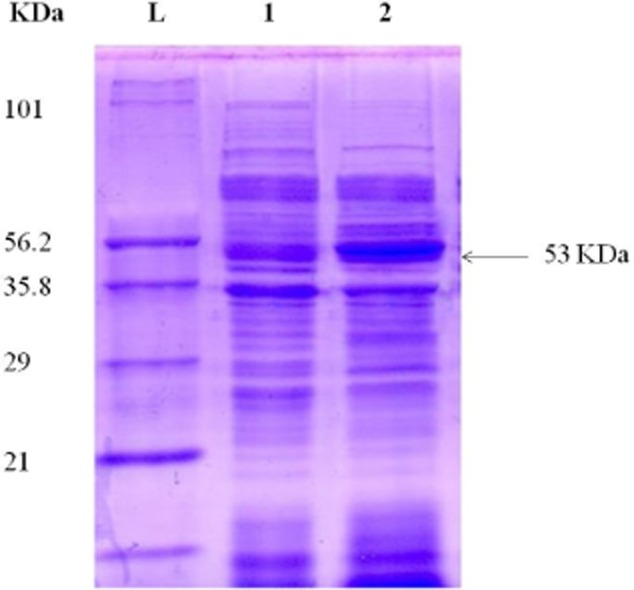
SDS-PAGE analysis of the overexpression of *GAD* gene by the recombinant *L. plantarum* Taj-Apis362: Lane L, marker proteins; Lane 1, extract of wild-type strain; Lane 2, cell extract of recombinant strain.

**Fig 3:**
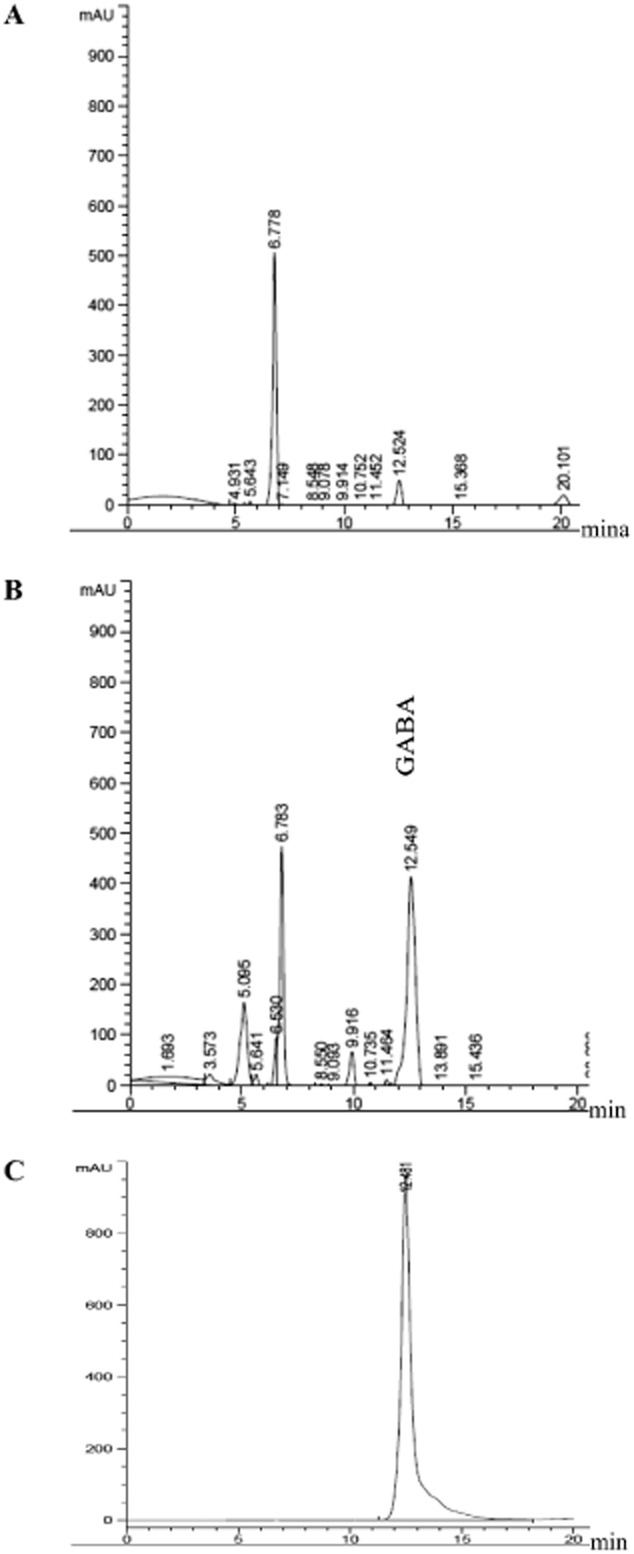
Representative chromatograms of GABA production.A. Wild type.B. Recombinant of *L. plantarum* Taj-Apis362. C. Standard GABA (5 mM).

The *L. plantarum* Taj-Apis362 *GAD* was expressed in the pMG36e vector as described in the *Materials and methods*, and the overexpression was monitored by SDS-PAGE. The SDS-PAGE analysis of extracted proteins showed one band of approximately 53 kDa which was strongly expressed in recombinant *L. plantarum* Taj-Apis362 harbouring pMG36e-*LbGAD* in comparison with the wild type of *L. plantarum* Taj-Apis362; though the expression levels of other proteins were almost same (Fig. 2). In order to confirm the presence of *L. plantarum* Taj-Apis362 *GAD* in *L. plantarum* Taj-Apis362 competent cells, the *GAD* activity of the cell extract was measured by high-performance liquid chromatography (HPLC), and the result was compared with GABA produced by the wild type. A noticeable peak was observed at a retention time of 12.52 min in the reaction products of wild-type *L. plantarum* Taj-Apis362 (Fig. 3A) and recombinant *L. plantarum* Taj-Apis362 (Fig. 3B) which were coincided with the peak of the GABA standard (Fig. 3C). It is apparent that *GAD* activity of the cell extract released by the recombinant (167.2 units/ml/min) was sevenfold greater than the *GAD* activity of wild-type cells (23.5 units/ml/min) confirming the presence of *LbGAD* gene encoded *GAD* with enhanced activity in the new host.

### Analysing and modeling

Preliminary experiments using *L. plantarum* Taj-Apis362 showed that the initial glutamic acid concentration, cultivation temperature, initial pH and incubation time were the most important factors for GABA production. To optimize the fermentation parameters for maximum GABA production, RSM was employed with the initial glutamic acid concentration set at 525 mM, cultivation temperature 37.5°C, initial pH 5.25 and incubation time 48 h in the central point (Table 1).

**Table 1:** Recombinant *L. plantarum* Taj-Apis362 treatment incorporations and responses

Trials	Factor A temperature (°C)	Factor B pH	Factor C glutamic acid (mM)	Factor D time (h)	Actual (GABA) (mM)	Predicted (GABA) (mM)	Absolute deviation
1	33.75	4.875	462.5	36	5.01	5.28	0.0543
2	41.25	4.875	462.5	36	5.23	5.02	0.0403
3	33.75	5.625	462.5	36	3.79	3.36	0.1145
4	41.25	5.625	462.5	36	5.81	6.42	0.1054
5	33.75	4.875	587.5	36	6.26	6.36	0.0161
6	41.25	4.875	587.5	36	6.18	5.64	0.0875
7	33.75	5.625	587.5	36	4.48	4.05	0.0960
8	41.25	5.625	587.5	36	6.42	6.64	0.0342
9	33.75	4.875	462.5	60	9.92	9.69	0.0223
10	41.25	4.875	462.5	60	6.44	6.72	0.0431
11	33.75	5.625	462.5	60	8.87	9.26	0.0438
12	41.25	5.625	462.5	60	9.71	9.61	0.0106
13	33.75	4.875	587.5	60	9.53	8.77	0.0801
14	41.25	4.875	587.5	60	4.90	5.33	0.0882
15	33.75	5.625	587.5	60	7.73	7.94	0.0270
16	41.25	5.625	587.5	60	8.25	7.82	0.0514
17	30	5.25	525	48	7.79	8.16	0.0465
18	45	5.25	525	48	7.99	7.78	0.0259
19	37.5	4.5	525	48	4.58	4.83	0.0550
20	37.5	6	525	48	5.50	5.40	0.0174
21	37.5	5.25	400	48	7.40	7.03	0.0494
22	37.5	5.25	650	48	5.81	6.34	0.0896
23	37.5	5.25	525	24	4.28	4.41	0.0301
24	37.5	5.25	525	72	9.98	10.01	0.0026
25	37.5	5.25	525	48	9.45	10.27	0.0864
26	37.5	5.25	525	48	10.40	10.27	0.0125
27	37.5	5.25	525	48	10.48	10.27	0.0204
28	37.5	5.25	525	48	10.60	10.27	0.0308
29	37.5	5.25	525	48	10.44	10.27	0.0158
30	37.5	5.25	525	48	10.25	10.27	0.0017

AAD = 3.869%, R^2^ = 0.972.

### Optimization by RSM

Fitting the data into different models (two factorial, cubic, linear and quadratic) and their subsequent analysis of variance (ANOVA) (Table 2) demonstrated that the quadratic model as shown in Eq. (1) was found to be the most suitable model to explain the influence of effective factors on GABA production.


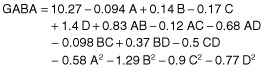
(1)

Where A is incubation temperature, B is initial pH; C is glutamic acid concentration, and D is incubation time.

**Table 2:** Analysis of variance for the regression of recombinant *L. plantarum* Taj-Apis362 harbouring pMG36e-*LbGAD*

Source	SS	DF	MS	*F*	*P*	
Model	141.86	14	10.13	37.68	0.0001	significant
A	0.21	1	0.21	0.79	0.3877	
B	0.49	1	0.49	1.83	0.1963	
C	0.73	1	0.73	2.73	0.1195	
D	46.95	1	46.95	174.57	0.0001	
AB	11.03	1	11.03	40.99	0.0001	
AC	0.22	1	0.22	0.81	0.3835	
AD	7.36	1	7.36	27.36	0.0001	
BC	0.15	1	0.15	0.57	0.4622	
BD	2.21	1	2.21	8.2	0.0118	
CD	4.04	1	4.04	15.03	0.0015	
A^2^	9.1	1	9.1	33.82	0.0001	
B^2^	45.6	1	45.6	169.53	0.0001	
C^2^	22.04	1	22.04	81.96	0.0001	
D^2^	16.08	1	16.08	59.79	0.0001	
Residual	4.03	15	0.27			
Lack of Fit	3.17	10	0.32	1.83	0.2615	not significant
Pure Error	0.87	5	0.17			
Cor Total	145.9	29				

A = temperature (°C), B = pH, C = glutamic acid (mM), D = time (h).

Analysis of variance (Table 2) showed the coefficient of determination of R^2^ = 0.972 with a very small ‘model *P* value’ (0.0001), high ‘lack of fit *P* value’ (0.2615) and adjusted coefficient of determination of R^2^ = 0.9465, reflecting that the model suitably represented the actual relations between the fermentation parameters selected. Both values (R^2^ adjusted and R^2^ predicted) are close to 1, which indicated an important correlation between the observed and predicted values. The adjusted value suggested that the total variation of 0.9465% predict is attributed to the independent variables, and just around 5.35 % of the whole variation cannot be described by the model. In addition for any terms in the model, a large *F* value and a small *P* value indicated a more significant effect on the respective response variables.

The ANOVA result showed that the GABA yield is mainly influenced by the terms D, AD, AB, BD, CD, A^2^, B^2^, C^2^ and D^2^ (Table 2). The most significant factor was D (time) with the *F* value of 174.57 and *P* value < 0.0001. The interactions of BC and AC factors were not significant. The four independent variables displayed quadratic effects on the responses. Although pH, temperature and glutamic acid were insignificant parameters (*P* value > 0.05), they had significant interaction with other parameters; therefore, they were utilized to develop the model (Table 2).

**Fig 4:**
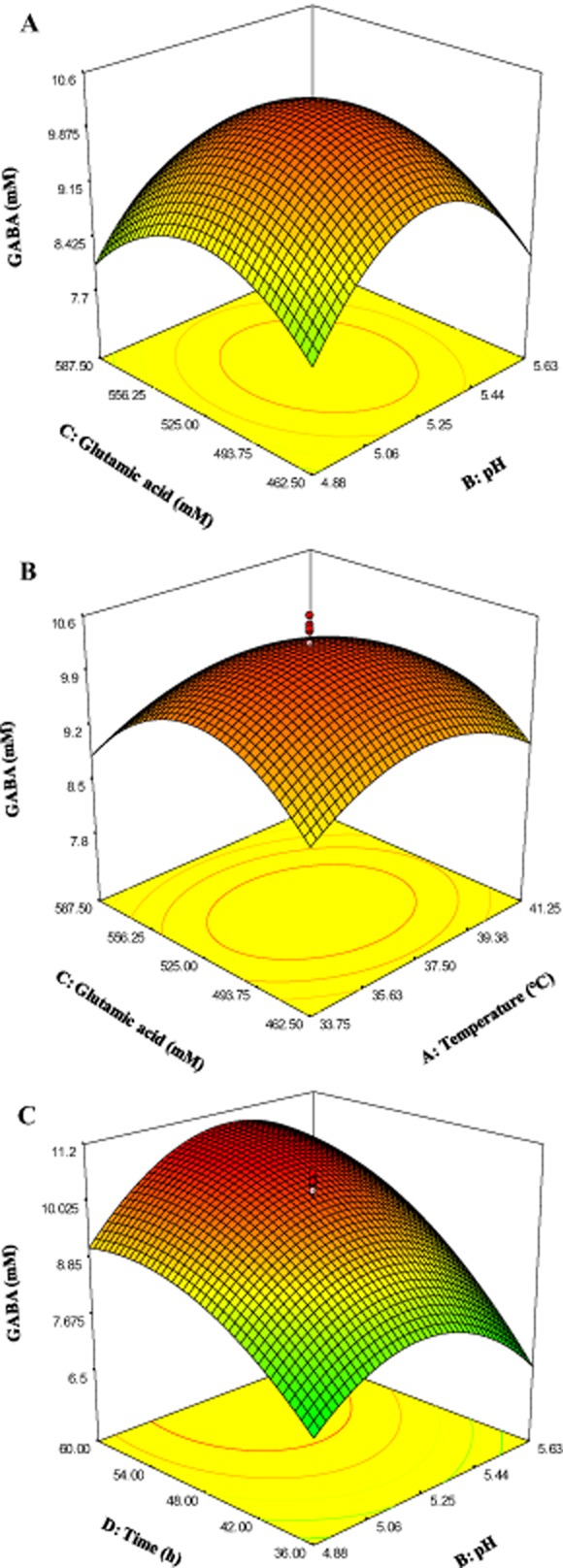
Three-dimensional surface plots showing the effect of different variables on GABA production.A. Effect of initial glutamic acid and pH.B. Effect of temperature and initial glutamic acid.C. Effect of pH and time.

Figure 4A shows the effect of glutamic acid and pH on GABA production when fixing the temperature (37.5°C) and retention time (48 h) at the central point. Gamma-aminobutyric acid production increased with the increase in pH and glutamic acid concentration up to pH 5.3 and 520 mM. As for the effects of temperature and glutamic acid concentration, GABA production was increased with the increase in both parameters up to 37.5°C and 525 mM, respectively (Fig. 4B), when fixing the pH at 5.25 and time at 48 h. It is apparent that high temperature and glutamic acid concentration suppressed GABA production. Figure 4C shows the effect of pH and incubation time on GABA production when fixing the initial glutamic acid concentration and incubation temperature at the central point (525 mM, 37.5°C). As can be seen, GABA concentration increased with the increase incubation time and pH up to 60 h and 5.25 respectively. However, GABA production decreased when the pH was increased to 5.63.

### Verification of the fitted model and optimum points

To verify the model, the actual values of GABA production by recombinant *L. plantarum* Taj-Apis362 were compared with the predicted values by computation of absolute average deviation (AAD) (Table 3). The computed AAD for this quadratic model was 0.8615%, indicated that the model equation was accurate and highly reliable. Moreover, to confirm the predicted optimum condition, the factor levels were set at the best values specified by the quadratic equation utilizing Design Expert software.

**Table 3:** Solution of optimum condition

No.	Temperature (°C)	pH	Glutamic acid (mM)	Time (h)	Actual (GABA) (mM)	Predicted (GABA) (mM)	Absolute deviation
1	36	5.31	497.973	60	11.09	11.23	0.01299
2	37	5.16	462.5	60	10.49	10.59	0.00972
3	37.5	5.31	514.883	48	9.72	9.70	0.00154
4	37.5	5.33	462.5	48	10.39	10.49	0.01021

R^2^ = 0.954, AAD = 0.8615%.

The optimum conditions for GABA production were predicted as shown in Table 3 together with their predicted and actual values. Maximum GABA production (11.23 mM) was obtained at the incubation temperature of 36°C, initial glutamic acid concentration of 497.973 mM, initial pH of 5.31 and incubation time of 60 h. The obtained experimental value of 11.09 mM was very close to the predicted value of 11.23 mM.

## Conclusion

A novel full-length *GAD* gene from a new GABA-producing microorganism, *L. plantarum* Taj-Apis362 was identified, cloned and successfully expressed in *L. plantarum* Taj-Apis362. The overexpression was confirmed by SDS-PAGE and *GAD* activity, which indicated 53 KDa protein and a significant increase in the GAD activity by more than sevenfold. The fermentation parameters were optimized using RSM. Under the optimum conditions with initial glutamic acid concentration 497.973 mM, cultivation temperature 36°C, initial pH 5.31 and incubation time 60 h, maximum GABA concentration was 11.09 mM, which is 1.55-fold higher than the optimized wild type. To our knowledge, this is the first report of cloning (clone-back) and overexpression of the *LbGAD* gene from *L. plantarum* to *L. plantarum* cells. These results suggest that this recombinant could be utilized for the industrial production of GABA as well as for the development of health foods rich in GABA.

## Experimental procedures

### Strains, plasmids and media

*Lactobacillus plantarum* Taj-Apis362 with high GABA-producing capacity was isolated from the honey stomach of honeybee *Apis dorsata* in Malaysia (Tajabadi *et al*., 2013) and used as the deoxyribonucleic acid (DNA) source and host for gene overexpression. Plasmid pTZ57R/T vector (MBI, Fermentas, USA) was used for DNA cloning and sequencing, and pMG36e (van de Guchte *et al*., 1989) vector for the overexpression of *LbGAD* protein. Competent cells were prepared according to a standard procedure with some modifications and stored at −80°C until use (Teresa Alegre *et al*., 2004). *Lactobacillus plantarum* Taj-Apis362 was cultured at 30°C in de Man, Rogosa and Sharpe (MRS) agar (Merck) supplemented with 5 μg/ml of erythromycin for the selection of transformants harbouring recombinant plasmids.

### PCR amplification of the full-length GAD gene and sequence analysis

Genomic DNA from *L. plantarum* Taj-Apis362 was purified and used as the template for the PCR analysis. Polymerase chain reaction amplification was performed using the designed forward (5′-atggcWatgttRtaYggWaaa-3′) and reverse (5′-ttagtgHgtgaaYccgtattt-3′) primers (Fan *et al*., 2012). Amplification by PCR was performed at 94°C for 3 min, 94°C for 40 s, 55°C for 1 min and 72°C for 2 min for 29 cycles using Pfu DNA polymerase (Fermentase, USA). The PCR product was purified using the Wizard SV Gel and PCR Clean Up Kit (Promega, Madison, Wi, USA). Then it was ligated into pTZ57R/T vector with T4 DNA ligase and transformed into *E. coli* TOP10 using a standard protocol (Sambrook *et al*., 2001). The positive transformants were selected on Luria-Bertani ampicillin plates, and several of them were confirmed by colony PCR using the same primer. The final validated positive clone of pTZ57R/T vector was sent to First Base Company (Malaysia) for sequence determination. Databases (GenBank) were searched for the similarity analysis of the *GAD* sequence obtained (http://blast.ncbi.nlm.nih.gov/Blast.cgi).

### Construction of pMG36e-LbGAD vector

The complete ORF region of *LbGAD* was amplified by PCR using pTZ57R/T-*LbGAD* as template and GADS58 as a forward primer (TAAGAGCTCTCATGGCA**ATG**TTGTATGG) (*Sac*I restriction site underlined and start codon in boldface) and GADX58 as a reverse primer (TAATCTAGATTAGTGTGTGAATCC) harbouring an *Xba*I site (underlined). The resulting 1410 bp PCR product (*LbGAD*) was purified using the Wizard DNA Purification Kit (Promega, Madison, WI) and digested with *Sac*I/*Xba*I. An approximately 1410 bp DNA was then inserted into the pMG36e expression vector to generate the recombinant expression vector construct pMG36e-*LbGAD*.

### Preparation of L. plantarum Taj-Apis362 competent cells

To prepare the *L. plantarum* Taj-Apis362 competent cells, the pre-cultured *L. plantarum* Taj-Apis362 in MRS medium was inoculated into the MRS medium, and grown at 30°C to an OD_600nm_ of 0.7–0.8. After cultivation, the cells were collected by centrifugation at 5000 × *g* for 2 min and washed with distilled water by centrifugation at 5000 × *g* for 2 min. The cells were washed with 10 mM MgCl_2_ solution (tow time), and finally suspended with electroporation buffer (0.5 M sucrose solution containing 10% glycerol).

### Overexpression of the LbGAD gene in L. plantarum Taj-Apis362

The prepared competent cells and plasmid pMG36e-*LbGAD* were mixed and transferred to a pre-chilled electroporation cuvette. Then they were exposed to a single electric pulse at 2.3 KV, 25 μF and 200 Ω using a Gene-Pulser (Bio-Rad, USA) according to Holo and Nes (1989). The suspensions were immediately mixed with 600 μl MRS broth containing 3% glycine, 5% sucrose, 20 mM MgCl_2_, and 2 mM CaCl_2_, and then incubated at 30°C for 2 h. The incubated cells were spread on the three MRS agar plates containing erythromycin (final concentration of 5 μg/ml) and incubated at 30°C for 48 h. Then positive transformants were selected on MRS agar erythromycin plates, and several of them were confirmed by colony PCR as well as restriction digestion of purified plasmid DNA with *EcoR*I and *Sac*I. The final validated positive purified plasmid was sent to First Base Company for sequence determination.

Recombinant *L. plantarum* Taj-Apis362 were cultivated in 50 ml MRS broth containing 5 μg/ml erythromycin at 30°C for 12 h. The cells were then centrifuged (11 000 × *g*, 4°C, 15 min), and the pellet were washed with distilled water (11 000 × *g*, 4°C, 15 min). Then the cells were re-suspended in PBS and disrupted by sonication [5 min (30 s on and 30 s off)]. The supernatant fraction was recovered by centrifugation at 11 000 × *g* for 15 min at 4°C. The supernatant was used for SDS-PAGE and the analysis of *GAD* activity. A colorimetric protein assay, based on the Bradford method, was used for the measurement of protein concentration (Bradford, 1976). This is a dye-binding assay based on the differential color change of Coomassie Brilliant blue G dye in response to various concentrations of protein. Thirty microlitre of the sample was mixed with 970 μl of Bradford reagent (Bio-Rad) in a 1.5 ml cuvette and mixed thoroughly. Incubated for 15 min in dark place at room temperature and absorbance was determined at OD_595_ using a spectrophotometer (Thermo Scientific, USA). The protein concentration was determined from the standard curve which was constructed by using various known concentration of BSA under the same procedure. All the samples which were used in *GAD* assay and SDS-PAGE were primarily standardized by aid of Bradford assay, and all total protein concentrations were the same. In addition, the samples run on the SDS-PAGE were total cell lysate.

### Analysis of GABA and GAD activity

Glutamate decarboxylase activity was measured by mixing the crude enzyme solution and 1.32 mM glutamic acid in 200 mM sodium acetate/acetic acid. The mixture was incubated in an incubator for 60 min at 37°C with 100 r.p.m., and the reaction was terminated by boiling. The amount of GABA produced was determined by HPLC (Agilent 1200 series HPLC system, Agilent Tech, Waldbonn, Germany) equipped with a Hypersil ODS C_18_ reverse-phase column with 5 μm diameter, 250 mm length and 4.6 mm internal diameter (Thermo. USA). A 100 μl culture broth filtered through a 0.22 μm filter, was derived, and the residue was dissolved in 20 μl of an ethanol-water-triethylamine solution (2:2:1) and evaporated by the vacuum pump to 300 millitorr, then 30 μl of an ethanol-water-triethylamine-phenylisothiocyanate solution (7:1:1:1) was added and incubated at room temperature for 20 min to allow the formation of phenylisothiocyanate-GABA and vacuumed again to 300 millitorr. After derivation, the sample was diluted and subjected to HPLC analysis. The injection volume was 20 μl with a flow rate of 0.6 ml/min.

The HPLC mobile phase A was a mixture prepared as follows: Sodium acetate three hydrates (10.254 g, 99%, A.C.S. reagent, Sigma-Aldrich) were dissolved in 900 ml deionized water. Then 500 μl triethylamine (Merck, Darmstadt, Germany) was added to the solution, which was rich in 1 l with water. The pH of the mobile phase A was adjusted to 5.8 using acetic acid (Merck, Darmstadt, Germany). High-performance liquid chromatography mobile phase B was acetonitrile (HPLC grade, Merck, Darmstadt, Germany) and mobile phase C was deionized water. All mobile phases were passed through a 0.22 μm membrane filter. The column temperature was set at 25°C. Sample injection volume was 20 μl, and the compound was detected through a diode array detector at 254 nm. The amount of GABA was calculated by comparing the peak area with the corresponding standard GABA. One unit of enzyme activity (U) was defined as the amount of enzymes that produced 1 μmol GABA/min.

## Experimental design and statistical analysis

### Experimental design

According to results of previous single-factor tests, a five-level four-variable central composite design (CCD) was employed in this study, resulting in 30 combinations (Table 1). Incubation temperature (30–45°C), initial pH (4.5–6), incubation time (24–72 h) and initial glutamic acid concentration (400–650 mM) were independent factors selected to optimize the GABA production by recombinant *L. plantarum* Taj-Apis362. To avoid bias, 30 treatments were carried out in a random order in which 24 axial points (treatment 1–24) and six center points (treatment 25–30) were considered (Table 1). Each experiment was performed in triplicate.

### RSM

The CCD experimental data were used for model fitting in RSM to find the best polynomial equation. These data were analysed and interpreted using Design Expert software (version 7.0 student trial, Stat Ease Inc. Minneapolis, USA). Three main analytical steps involving ANOVA, a regression analysis and the plotting of response surface were performed to establish an optimum condition for GABA production. Then, the predicted values obtained from RSM model were compared with the actual values for testing the model. Finally, the experimental values of predicted optimal conditions (Table 3) were used as validating set and compared with the predicted values.

### Verification of estimated data

To test the estimation capabilities of the technique, the estimated responses obtained from RSM were compared with the observed responses using the coefficient of determination (R^2^) and absolute average deviation (AAD). The R^2^ and AAD were calculated by the following equations.


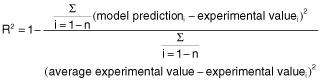
(2)

When the *n* is the number of experimental data.


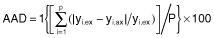
(3)

Where y_i, ex_ and y_i, ax_ are the experimental and calculated responses, respectively, and *p* is the number of the experimental run.

R^2^ is a factor that showed the reduction amount in the variability of response obtained by using the repressor variables in the model. Because R^2^ alone is not a measure of the model accuracy, it is necessary to use AAD analysis, which is a direct method for describing the deviations. In addition, evaluation of R^2^ and AAD values together showed the accuracy of the model. However, R^2^ must be close to 1.0, and the AAD between the predicted and observed data must be as small as possible. The acceptable values of R^2^ and AAD values mean that the model equation defines the true behaviour of the system, and it can be used for interpolation in the experimental domain (Bas and Boyaci, 2007).

## Conflict of interest

None declared.
